# Supplementary material for: Effects of deconstructed Tai Chi step training combined with conventional rehabilitation on lower limb function in brunnstrom stage III stroke patients: A randomized controlled trial
Source: PLoS One. 2026 May 7;21(5):e0348765. doi: 10.1371/journal.pone.0348765 (PMC13152142; doi:10.1371/journal.pone.0348765)
Supplement: S2 File — (DOCX) [file pone.0348765.s002.docx]

**Trial Study Protocol**

**Title:**
Deconstructed Tai Chi Step Training Combined with Conventional Rehabilitation for Lower Limb Function Improvement in Brunnstrom Stage III Stroke Patients: A Randomized Controlled Trial

**Investigators:**

- Jinxin Chang (Principal Investigator), School of Physical Education, Shanxi University, China
- Pengcheng Qu, Chinese Martial Arts Academy, Beijing Sport University, China
- Yindong Li (Corresponding Author), Chinese Martial Arts Academy, Beijing Sport University, China

**1. Background and Objectives**

**Background:**
Stroke survivors in Brunnstrom Stage III face significant challenges due to extensor spasticity and limited joint isolation, hindering functional recovery. Conventional rehabilitation often lacks targeted strategies for this stage. Tai Chi, with its emphasis on mind-body coordination, may address these gaps but requires adaptation for Stage III patients.

**Primary Objective:**
To evaluate the efficacy of a deconstructed Tai Chi step protocol combined with conventional rehabilitation in improving lower limb motor function, walking ability, and joint range of motion (ROM) in Brunnstrom Stage III stroke patients.

**Secondary Objectives:**

1. Compare outcomes between Tai Chi-based intervention and conventional rehabilitation + limb synergy training.
2. Assess the feasibility and safety of the Tai Chi protocol in clinical settings.

**2. Methods**

**2.1 Study Design**

- **Design:** Parallel-group, single-center, randomized controlled trial (RCT).
- **Duration:** 8-week intervention with pre- and post-assessment.
- **Randomization:** 1:1 allocation using computer-generated block randomization.

**2.2 Participants**

**Inclusion Criteria:**

1. Confirmed ischemic/hemorrhagic stroke (≤6 months post-onset).
2. Brunnstrom Stage III for lower limbs (active hip/knee flexion in sitting/standing, peak extensor spasticity).
3. Age 30–70 years, stable vital signs, MMSE ≥24.
4. No severe joint deformities or comorbidities.

**Exclusion Criteria:**

1. Severe cognitive impairment (MMSE <24).
2. Uncontrolled hypertension (BP ≥180/100 mmHg).
3. Inability to complete ≥80% of sessions.

**Sample Size:**

- Calculated using G*Power 3.1 (α = 0.05, power = 0.80, effect size d = 1.2).
- Initial recruitment: 52 participants (26 per group), accounting for 20% attrition.

**2.3 Interventions**

**Both Groups Received:**

- **Conventional Rehabilitation (60 min/day, 5 days/week):**
  - Physical therapy: Medium-frequency electrical stimulation (20 min), limb linkage exercises (10 min).
  - Traditional therapy: Massage (15 min), acupuncture at ST36/GB34 (15 min).

**Experimental Group (E Group):**

- **Deconstructed Tai Chi Step Training (40 min/day, 5 days/week):**
  1. **Warm-up (15 min):**
     - *Zhan Zhuang* (standing meditation): 30° knee flexion, 1.5–3 min/set.
     - *Du Li Bu* (single-leg stance): 10 repetitions/set.
  2. **Core Training (20 min):**
     - *Ce Xing Bu* (lateral stepping): 45° hip abduction, 5–8 steps/set.
     - Forward/backward stepping: Hip/knee flexion ≥60°, 5 steps/set.
  3. **Cool-down (5 min):** Static stretching.
  4. **Intensity:** Target heart rate = 40%–60% max HR (220 − age).

**Control Group (C Group):**

- **Limb Synergy Training (40 min/day, 5 days/week):**
  1. **Slope Standing Training (20 min):** 30°–60° inclination.
  2. **Limb Coordination Training (10 min × 2 sets):** Bilateral flexion/extension using a four-limb linkage trainer.

**2.4 Outcome Measures**

**Primary Outcome:**

- **Fugl-Meyer Assessment for Lower Extremity (FMA-LE):** Total score (0–34).

**Secondary Outcomes:**

1. **Holden Walking Ability Classification:** Grades I–III.
2. **Joint ROM:** Hip (flexion, extension, abduction), knee (flexion, rotation), ankle (dorsiflexion, plantarflexion, inversion/eversion).

**Safety Monitoring:**

- Blood pressure, heart rate, adverse events (e.g., falls, joint sprains).

**2.5 Data Collection and Analysis**

**Data Collection:**

- Baseline and post-intervention assessments by blinded evaluators.
- Real-time wireless motion capture system (FAB) for ROM measurements.

**Statistical Analysis (SPSS 26.0):**

- **Within-group:** Paired t-tests.
- **Between-group:** Independent t-tests (parametric) or Mann-Whitney U tests (non-parametric).
- **Effect Size:** Cohen’s d for FMA-LE.

**2.6 Ethical Considerations**

- Written informed consent obtained.
- Adverse events reported to the ethics committee.
- Data anonymized and stored securely.

**2.7 Timeline**

- **Recruitment:** September 10–November 1, 2022.
- **Intervention:** November 1, 2022–January 31, 2023.
- **Analysis:** February 2023.
