# Supplementary material for: Effects of deconstructed Tai Chi step training combined with conventional rehabilitation on lower limb function in brunnstrom stage III stroke patients: A randomized controlled trial
Source: PLoS One. 2026 May 7;21(5):e0348765. doi: 10.1371/journal.pone.0348765 (PMC13152142; doi:10.1371/journal.pone.0348765)
Supplement: Attachment — Submitted filename: Comments.docx [file pone.0348765.s003.docx]

Overall Evaluation

The topic is clinically relevant, addressing a significant gap in stage-specific post-stroke rehabilitation. The findings are promising and supported by quantitative evidence. However, several aspects, particularly in methodological transparency, discussion depth, and theoretical contextualization require clarification or strengthening to enhance scientific rigor and reproducibility.

Comments

The concept of “deconstructed Tai Chi” tailored to Brunnstrom Stage III is original and aligns with growing interest in culturally adapted, low-cost rehabilitation methods. However, the degree of novelty should be clarified in relation to prior studies on simplified Tai Chi interventions in stroke recovery (e.g., Li et al., 2018; Zou et al., 2019).

The introduction could better distinguish this protocol from prior modified Tai Chi programs by emphasizing how joint isolation targeting extensor synergy specifically meets the neurophysiological needs of Stage III patients. Add 2–3 sentences in the introduction explaining how deconstructed Tai Chi differs in biomechanical intent and progression from previously reported Tai Chi-based interventions for post-stroke recovery.

The introduction currently introduces stroke rehabilitation but lacks sufficient pathophysiological context linking vascular vulnerability and metabolic risk factors to the need for rehabilitative intervention. Add below phrase with recent literature support as shown to strengthens the scientific justification for this study.

“Previous research has emphasized that cerebrovascular fragility and metabolic risk factors, particularly obesity, play a pivotal role in precipitating fatal brain hemorrhages even in individuals without prior clinical symptoms [Sheikh et al., 2025, <https://pmc.ncbi.nlm.nih.gov/articles/PMC11875705/> ]. These findings underscore the importance of preventive and restorative strategies aimed at enhancing neurovascular stability and functional resilience, which are core objectives guiding rehabilitation approaches in stroke and related neurological disorders.”

Methodology

Describe how random allocation was performed (e.g., sealed envelopes, computer-generated sequence) and who managed allocation concealment.

While blinding therapists is difficult, please elaborate on how assessor blinding integrity was verified.

Clarify the reference study used for the effect size (d = 1.2). A citation and calculation example would strengthen credibility.

Report dropout reasons (if any) and whether intention-to-treat (ITT) analysis was conducted.

Include a methodological clarification paragraph specifying randomization control, adherence verification, and bias minimization.

Results

The clinical relevance of the between-group difference in FMA-LE (+5.6 vs. +1.8) should be interpreted in the context of minimal clinically important difference (MCID) for post-stroke lower limb function. Additionally, the use of multiple parametric tests across ROM and FMA variables without correction (e.g., Bonferroni) increases the risk of Type I error. Discuss the clinical meaningfulness of the effect size and specify whether adjustments for multiple comparisons were applied.

Discussion

The discussion appropriately highlights neuroplastic and proprioceptive mechanisms; however, it misses deeper integration with motor control theory and neurorehabilitation frameworks.

The section could benefit from citing functional imaging or EMG-based Tai Chi studies to support claims about cortical reorganization.

The discussion currently emphasizes clinical rehabilitation outcomes but misses integration with recent multidisciplinary evidence linking physical activity to neurobiological recovery mechanisms. Add below phrase to strengthen holistic model where physical therapy not only restores motor function but also enhances neuroplasticity, emotional resilience, and overall brain health.

“Emerging evidence has demonstrated that structured physical therapy and exercise interventions can positively modulate neurobiological pathways, promoting neurogenesis, mood stabilization, and functional recovery in neurological disorders [Nasif et al., 2025, <https://doi.org/10.31579/2578-8868/359> ]. These findings highlight the broader rehabilitative potential of physical engagement in restoring neurovascular and psychosocial integrity following brain injury or hemorrhage, thereby supporting integrated rehabilitation frameworks for stroke survivors.”

Limitations and Future Scope

Include potential gender imbalance or age-related variability in recovery. Discuss cultural transferability — can this Tai Chi-based model be applied outside East Asia? Consider proposing longitudinal follow-up or home-based adaptation as a research priority. Add one paragraph proposing multicenter validation and wearable-based outcome tracking (e.g., kinematic or EMG-based monitoring) to enhance reproducibility and global applicability.
