# Supplementary material for: Effects of deconstructed Tai Chi step training combined with conventional rehabilitation on lower limb function in brunnstrom stage III stroke patients: A randomized controlled trial
Source: PLoS One. 2026 May 7;21(5):e0348765. doi: 10.1371/journal.pone.0348765 (PMC13152142; doi:10.1371/journal.pone.0348765)
Supplement: Attachment — Submitted filename: Response to Reviewers.docx [file pone.0348765.s004.docx]

**Response to Reviewers**

**Manuscript ID: PONE-D-25-27347**
**Title:** Effects of Deconstructed Tai Chi Step Training Combined with Conventional Rehabilitation on Lower Limb Function in Brunnstrom Stage III Stroke Patients: A Randomized Controlled Trial

Dear Academic Editor and Reviewers,

We sincerely thank you for your careful evaluation of our manuscript and for the constructive comments and suggestions. We have revised the manuscript extensively in response to the editor’s and reviewers’ recommendations. Major revisions include clarification of the study rationale and stage-specific innovation, improvement of methodological transparency, addition of explicit outcome and statistical analysis subsections, refinement of the Results presentation, strengthening of the Discussion, updating of the reference list, and moderation of the overall tone of the manuscript. The revised manuscript has been carefully checked for clarity and internal consistency.

Below, we provide a point-by-point response.

**Note:** Locations refer to section numbers in the revised manuscript.

**Responses to Journal Requirements and Editorial Comments**

**Journal Requirement 1**

Comment: Please ensure that your manuscript meets PLOS ONE style requirements, including file naming.

Response:
Thank you for this reminder. We have revised the manuscript to better align with PLOS ONE style expectations, including the title, abstract structure, section organization, and figure/table presentation. We will also ensure that the final uploaded files are named and formatted according to the journal requirements at the time of resubmission.

**Journal Requirement 2**

Comment: Please provide trial registry information and the complete date range for recruitment and follow-up.

Response:
We have revised the manuscript to provide the full name of the registry and the registration number on the title page and in the Methods section. We now state that the trial was registered in the International Traditional Medicine Clinical Trial Registry (ITMCTR; a WHO ICTRP Primary Registry) under registration number ITMCTR2025000972. We have also added the date range for participant recruitment and the date range for intervention and outcome assessment in Section 2.1.

**Journal Requirement 3**

Comment: Please ensure that all raw data required to replicate the study findings are fully available.

Response:
We appreciate the importance of this requirement. In the current revised manuscript, the Data Availability Statement remains provisional. Before final resubmission, we will update this statement to comply with the PLOS ONE minimal dataset policy and will provide the de-identified underlying data required to reproduce the reported findings, including the values underlying the tables and figures.

**Journal Requirement 4**

Comment: Please ensure that the corresponding author’s ORCID iD is validated in Editorial Manager.

Response:
Thank you. The corresponding author will complete ORCID validation in Editorial Manager before resubmission.

**Journal Requirement 5**

Comment: Please include captions for Supporting Information files at the end of the manuscript.

Response:
Thank you for this important reminder. We will add the captions for all Supporting Information files at the end of the manuscript and ensure that the in-text citations are updated accordingly before final resubmission.

**Journal Requirement 6**

Comment: Please review and evaluate the publications suggested by reviewers and cite them if relevant.

Response:
We have carefully reviewed the suggested literature and incorporated relevant references where they strengthened the rationale, methodological background, and discussion of mechanisms and rehabilitation implications. We did not add suggested references mechanically; instead, we selectively included those that were directly relevant to the scope and focus of the present study.

**Additional Editor Comment**

Comment: The study is clinically relevant and innovative, but several sections require clarification to strengthen methodological rigor, theoretical grounding, and interpretive depth.

Response:
We appreciate this overall assessment. In response, we revised the Introduction to better define the stage-specific novelty of the deconstructed Tai Chi protocol, expanded the Methods to clarify randomization, concealment, blinding, attrition, and analysis population, revised the Results to improve statistical reporting, and strengthened the Discussion by integrating motor control theory, rehabilitation frameworks, updated references, and a more balanced interpretation of the findings.

**Response to Reviewer 1**

**Comment 1**

I suggest adding a subsection to describe the outcomes of interest.

Response:
Thank you for this suggestion. We have added a dedicated subsection, **“**2.4 Outcome Measures,” which clearly describes the primary and secondary outcomes, the measurement tools, and the timing of assessments.
Location: Section 2.4.

**Comment 2**

I suggest stating when the participant in the E group discontinued the intervention and when the participant in the C group lost to follow-up.

Response:
We have added the specific time points and reasons. One participant in the experimental group discontinued the intervention at week 4 because of family obligations, and one participant in the control group was lost to follow-up after week 6 and could not be contacted.
Location: Section 2.2.2

**Comment 3**

The sample size was calculated based on a continuous outcome. In this manuscript, five outcomes were assessed. Please include potential inflated type I error as a limitation.

Response:
We agree. We have addressed this point in two places. In Section 2.5, we now state that no formal adjustment for multiple comparisons was applied and that the secondary ROM analyses should be interpreted cautiously. We also added this issue explicitly to the Limitations section.
Location: Sections 2.5 and 4.4.

**Comment 4**

The exclusion was post-randomization and made the analysis per-protocol.

Response:
We agree and have clarified this point. The revised manuscript now distinguishes pre-randomization exclusions from post-randomization discontinuations and explicitly states that the final analysis was conducted on a per-protocol basis. We also state that intention-to-treat analysis was not performed.
Location: Sections 2.2.2 and 2.5.

**Comment 5**

Please include the timing when the outcomes were assessed.

Response:
We have added this information explicitly. The revised manuscript now states that outcome measures were assessed at baseline before randomization and again immediately after completion of the 8-week intervention period.
Location: Section 2.4.

**Comment 6**

Please state the primary population of analysis (e.g., intention-to-treat, per-protocol, etc.).

Response:
We have now clearly stated that the primary analysis was conducted on a per-protocol basis.
Location: Sections 2.2.2 and 2.5.

**Comment 7**

Please revise the sentence to clarify that both t-test and Mann–Whitney U test are for continuous variables, but one is parametric and the other is non-parametric.

Response:
We have revised the Statistical Analysis section to clarify that independent t-tests were used for normally distributed continuous variables, whereas Mann–Whitney U tests were used for non-normally distributed variables and for ordinal data where appropriate.
Location: Section 2.5.

**Comment 8**

Please clarify whether the between-group comparisons were based on pre-and-post change or post-intervention measurements.

Response:
We have explicitly stated that between-group comparisons for continuous outcomes were based on change scores (post-intervention minus baseline).
Location: Section 2.5.

**Comment 9**

Baseline characteristics were expected to be balanced due to the randomization. Please remove the Statistic and p-value columns.

Response:
We agree. Table 1 has been revised to present baseline characteristics descriptively only. The Statistic and p-value columns were removed, and the corresponding Results text was revised accordingly.
Location: Section 3.1 and Table 1.

**Comment 10**

Please do not report p-values if those comparisons were not the primary objective of the study. Reporting means and 95% confidence intervals would be sufficient.

Response:
We appreciate this suggestion. We revised the Results section to emphasize descriptive changes, 95% confidence intervals, and effect sizes for the major outcomes. We also reduced overreliance on repeated significance statements, particularly in the presentation of the continuous outcomes.
Location: Sections 3.2–3.4.

**Comment 11**

A binary outcome was not considered when calculating the required sample size.

Response:
Thank you. We have clarified in Section 2.2.1 that the sample size calculation was based on the primary continuous outcome. We also acknowledge in the Discussion/Limitations that additional outcomes, including Holden classification, were analyzed beyond the outcome used for sample size estimation.
Location: Sections 2.2.1 and 4.4.

**Response to Reviewer 2**

**Comment**

Please ensure that all references are thoroughly incorporated and updated to reflect the latest developments in the field.

Response:
Thank you for this helpful recommendation. We reviewed and updated the reference list, particularly in the Introduction and Discussion. The revised manuscript now includes additional recent studies where relevant to strengthen the background, methodological framing, and discussion of mechanisms and rehabilitation implications.
Location: Introduction, Discussion, and References.

**Response to Reviewer 3**

**Comment**

The study addresses a pertinent clinical question with a robust methodological approach.

Response:
We sincerely thank the reviewer for this positive and encouraging evaluation of our work.

**Response to Reviewer 4**

**Comment 1**

The novelty of “deconstructed Tai Chi” should be clarified in relation to prior studies on simplified Tai Chi interventions in stroke recovery.

Response:
We appreciate this comment and revised the Introduction accordingly. The revised text now distinguishes the present protocol from previously reported modified or simplified Tai Chi interventions by emphasizing that our approach was specifically designed for the motor characteristics of Brunnstrom Stage III, including extensor synergy dominance, limited selective joint control, and impaired weight shifting. We also clarify that the present protocol reorganizes training according to stage-specific biomechanical and neurophysiological demands rather than merely reducing movement complexity.
Location: Introduction.

**Comment 2**

The Introduction lacks sufficient pathophysiological context.

Response:
Thank you. Rather than adding the suggested wording verbatim, we strengthened the Introduction in a way that remained closely aligned with the focus of our study. Specifically, we added text emphasizing disrupted neuromuscular coordination, impaired postural control, limited weight transfer, and the relevance of targeted intervention during the subacute stage of recovery.
Location: Introduction.

**Comment 3**

Describe how random allocation was performed and who managed allocation concealment.

Response:
We have revised Section 2.1 to state that random allocation was performed using a computer-generated random sequence with a block size of 4, and that the allocation sequence was concealed in sequentially numbered, opaque, sealed envelopes prepared and managed by an independent researcher not involved in recruitment, intervention delivery, or outcome assessment.
Location: Section 2.1.

**Comment 4**

Please elaborate on how assessor blinding integrity was verified.

Response:
We expanded Section 2.2.3. The revised manuscript now states that after each evaluation session, assessors were asked to guess group assignment, and that blinding was considered successful if the correct guess rate did not exceed chance level. The observed guess accuracy was 38%, suggesting that assessor blinding was likely maintained throughout the study.
Location: Section 2.2.3.

**Comment 5**

Clarify the reference study used for the effect size (d = 1.2).

Response:
We clarified in Section 2.2.1 that the sample size calculation was based on the primary continuous outcome and used an assumed effect size of d = 1.2, derived from the between-group difference reported in Zhao et al.
Location: Section 2.2.1.

**Comment 6**

Report dropout reasons and whether ITT analysis was conducted.

Response:
This information has now been added to Section 2.2.2. We report the timing and reasons for the two post-randomization losses and explicitly state that the final analysis was conducted on a per-protocol basis and that intention-to-treat analysis was not performed.
Location: Section 2.2.2.

**Comment 7**

Include a methodological clarification paragraph specifying randomization control, adherence verification, and bias minimization.

Response:
We addressed this request by revising Sections 2.1, 2.2.3, and 2.3.4. These revisions clarify random sequence generation and concealment, adherence monitoring using attendance logs, standardized therapist training, scripted intervention delivery, and blinded outcome assessment.
Location: Sections 2.1, 2.2.3, and 2.3.4.

**Comment 8**

Interpret the clinical relevance of the between-group FMA-LE difference in the context of MCID, and clarify whether multiple-comparison adjustment was applied.

Response:

We appreciate this important point. In the revised manuscript, we added 95% confidence intervals and effect sizes for the major outcomes and discussed the potential clinical relevance of the FMA-LE difference more cautiously in the Discussion. We also clarified in Section 2.5 that no formal adjustment for multiple comparisons was applied and that the secondary ROM analyses should therefore be interpreted cautiously. This issue is also acknowledged in the Limitations section.

Location: Sections 2.5, 4.2, and 4.4.

**Comment 9**

The Discussion should better integrate motor control theory and neurorehabilitation frameworks.

Response:
Thank you. We substantially revised Section 4.1 to incorporate motor control theory, task-specific repetition, progressive joint dissociation, controlled weight transfer, and a motor relearning framework.
Location: Section 4.1.

**Comment 10**

The Discussion could benefit from citing functional imaging or EMG-based Tai Chi studies to support claims about cortical reorganization.

Response:
We appreciate this suggestion. While we did not identify a functional imaging study that matched our protocol and patient stage closely enough to cite directly, we strengthened the mechanistic discussion by adding updated EMG-/mechanistic-/neuromodulation-related references and by clarifying that no neurophysiological measures were collected in the present study. This limitation is now explicitly stated.
Location: Sections 4.1 and 4.4.

**Comment 11**

Please integrate broader evidence linking physical activity to neurobiological recovery.

Response:
We revised the Discussion to broaden the interpretation of the intervention beyond biomechanical practice alone. The revised text now notes that structured exercise may contribute to neurobiological recovery processes relevant to stroke rehabilitation and integrates this idea into the overall interpretation of the findings.
Location: Sections 4.1 and 4.3.

**Comment 12**

Please expand the limitations and future directions.

Response:
We revised Section 4.4 to address this comment directly. The revised section now includes the small sample size, per-protocol analysis, lack of neurophysiological assessment, possible performance bias, age- and sex-related variability, cultural transferability, home-based adaptation, multicenter validation, and the future use of wearable sensors, gait kinematics, and surface EMG.
Location: Section 4.4.

**Response to Reviewer 5**

**Comment 1**

Avoid repetitive “significantly improves” phrasing; maintain scientific neutrality.

Response:
We revised the title, abstract, and conclusion to adopt a more neutral tone. Expressions such as “significantly improves” and other stronger promotional language were replaced with more cautious formulations such as “was associated with greater improvement” and “may represent a practical adjunct.”
Location: Title, Abstract, and Conclusion.

**Comment 2**

Add explicit hypotheses.

Response:
We added an explicit hypothesis statement at the end of the Introduction. The revised text now states that we hypothesized that deconstructed Tai Chi step training combined with conventional rehabilitation would produce greater improvements in lower limb motor function, walking ability, and joint mobility than conventional rehabilitation plus limb synergy training.
Location: Introduction.

**Comment 3**

Provide numerical justification for the sample size calculation.

Response:
We revised Section 2.2.1 to state that the sample size was calculated using G*Power 3.1, with α = 0.05, power = 0.80, and an assumed effect size of d = 1.2 derived from Zhao et al. We also report that this yielded a required minimum of 20 participants per group and that 52 participants were initially recruited to account for anticipated attrition.
Location: Section 2.2.1.

**Comment 4**

Include CI and η² effect sizes.

Response:
We revised the Statistical Analysis and Results sections to report 95% confidence intervals and effect sizes for the major findings. The revised manuscript now includes Cohen’s d for the between-group FMA-LE difference and partial η² for the repeated-measures ANOVA.
Location: Sections 2.5 and 3.2–3.4.

**Comment 5**

In the Discussion, cite neuroplasticity mechanisms with updated evidence.

Response:
We revised the Discussion to incorporate updated references and a more explicit mechanistic framework, including motor control, neural adaptation, proprioceptive engagement, and movement reorganization.
Location: Section 4.1.

**Comment 6**

Clarify randomization and concealment methods.

Response:
As noted above, Section 2.1 has been revised to describe the computer-generated random sequence, block size, sealed-envelope concealment procedure, and the role of the independent researcher managing allocation.
Location: Section 2.1.

**Comment 7**

Include or re-analyze data with repeated-measures ANOVA or mixed models.

Response:
We revised the Statistical Analysis section and restructured the Results accordingly. A 2 × 2 repeated-measures ANOVA (group × time) was used for FMA-LE and ROM outcomes to assess time, group, and interaction effects.
Location: Sections 2.5, 3.2, and 3.4.

**Comment 8**

Report 95% CI for all major results.

Response:
We added 95% confidence intervals for the major continuous results, including the between-group difference in change scores for FMA-LE and the reported change estimates for ROM outcomes.
Location: Sections 3.2–3.4.

**Comment 9**

Improve Discussion balance and moderate the tone of conclusions.

Response:
We revised the Discussion and Conclusion to reduce overstatement and improve balance. The revised manuscript now interprets the findings within a stage-specific rehabilitation context, acknowledges methodological limitations more explicitly, and avoids broad claims of superiority beyond the scope of the present study.
Location: Sections 4 and 5.

**Comment 10**

Update literature (2024–2025) and improve figure reporting.

Response:
We updated the reference list with additional recent studies where relevant. We also revised the figure captions to clarify that the data are presented as mean ± SD and to standardize the significance notation.
Location: References and figure legends.

We hope that these revisions have addressed the reviewers’ and editor’s concerns and have substantially improved the manuscript. We are grateful for the constructive feedback and appreciate the opportunity to revise our work.

Sincerely,
Yindong Li
On behalf of all authors
